# Predictive Power of a Body Shape Index for Development of Diabetes, Hypertension, and Dyslipidemia in Japanese Adults: A Retrospective Cohort Study

**DOI:** 10.1371/journal.pone.0128972

**Published:** 2015-06-01

**Authors:** Misuzu Fujita, Yasunori Sato, Kengo Nagashima, Sho Takahashi, Akira Hata

**Affiliations:** 1 Chiba University, Department of Public Health, Chiba City, Chiba, Japan; 2 Chiba University, Hospital Clinical Research Center, Chiba City, Chiba, Japan; Weill Cornell Medical College in Qatar, QATAR

## Abstract

**Background/Objectives:**

Recently, a body shape index (ABSI) was reported to predict all-cause mortality independently of body mass index (BMI) in Americans. This study aimed to evaluate whether ABSI is applicable to Japanese adults as a predictor for development of diabetes, hypertension, and dyslipidemia.

**Subjects/Methods:**

We evaluated the predictive power of ABSI in a retrospective cohort study using annual health examination data from Chiba City Hall in Japan, for the period 2008 to 2012. Subjects included 37,581 without diabetes, 23,090 without hypertension, and 20,776 without dyslipidemia at baseline who were monitored for disease incidence for 4 years. We examined the associations of standardized ABSI, BMI, and waist circumference (WC) at baseline with disease incidence by logistic regression analyses. Furthermore, we conducted a case-matched study using the propensity score matching method.

**Results:**

Elevated BMI, WC, and ABSI increased the risks of diabetes and dyslipidemia [BMI-diabetes: odds ratio (OR) = 1.26, 95% confidence interval (95%CI) = 1.20−1.32; BMI-dyslipidemia: OR = 1.15, 95%CI = 1.12−1.19; WC-diabetes: OR = 1.24, 95%CI = 1.18−1.31; WC-dyslipidemia: OR = 1.15, 95%CI = 1.11−1.19; ABSI-diabetes: OR = 1.06, 95%CI = 1.01−1.11; ABSI-dyslipidemia: OR = 1.04, 95%CI = 1.01−1.07]. Elevated BMI and WC, but not higher ABSI, also increased the risk of hypertension [BMI: OR = 1.32, 95%CI = 1.27−1.37; WC: OR = 1.22, 95%CI = 1.18−1.26; ABSI: OR = 1.00, 95%CI = 0.97−1.02]. Areas under the curve (AUCs) in regression models with ABSI were significantly smaller than in models with BMI or WC for all three diseases. In case-matched subgroups, the power of ABSI was weaker than that of BMI and WC for predicting the incidence of diabetes, hypertension, and dyslipidemia.

**Conclusions:**

Compared with BMI or WC, ABSI was not a better predictor of diabetes, hypertension, and dyslipidemia in Japanese adults.

## Introduction

Obesity is a well-known risk factor for mortality from all causes [1, 2] and cardiovascular disease (CVD) [1, 3], and for incidences of non-communicable diseases such as diabetes [4, 5], hypertension [5, 6], and dyslipidemia [5]. Although body mass index (BMI, weight in kilograms divided by the square of height in meters) is widely used as a measure of obesity, it has potential weaknesses. The BMI does not distinguish weight due to fat accumulation from muscle weight [7, 8] and does not distinguish peripheral fat from abdominal fat [9], the latter being more strongly associated with CVD risk. To partly overcome these weaknesses, waist circumference (WC) is used as an indicator of abdominal fat accumulation, and many studies have shown that WC can predict mortality from all causes and CVD more accurately than BMI [10, 11]. However, WC reflects not only abdominal fat accumulation but also overall body size (height and weight). In fact, WC is strongly correlated with both weight and BMI [12, 13].

Krakauer et al. described a new anthropometric measure, a body shape index (ABSI), which quantifies abdominal adiposity relative to BMI and height, and reported that ABSI predicted all-cause mortality independently of BMI in a large cohort of American adults during an average 4.8-year follow-up [13]. They also indicated that the predictive power of ABSI for all-cause mortality differed among ethnicities, with less predictive power in Latinos than Caucasian and African Americans, and acknowledged that additional cohort studies with other ethnic groups are necessary to clarify the limits of ABSI predictive efficacy [13]. To date, six cohort studies evaluating the ABSI for prediction of mortality or morbidity have been published [14–19]. Of these, three described the predictive power of ABSI for mortality from CVD [14] and all causes [15, 16]. The largest of these studies, including 46,651 Europeans, concluded that the predictive powers of several abdominal obesity indicators, such as WC, waist-to-hip ratio, and waist-to-stature ratio, were stronger predictors of CVD mortality than were BMI and ABSI [14], while a British study of 7,011 adults reported that ABSI was a robust predictor of all-cause mortality [15]. The smallest study, with 142 hemodialysis patients, found no relation between ABSI and mortality [16]. In the remaining three studies, the principle outcome measure was morbidity. One showed that ABSI was significantly associated with total stroke incidence in men, while BMI was not [17]. The others found that ABSI was associated with development of diabetes [18] or hypertension [19], although the predictive power was no better than WC or BMI.

The aim of this study is to evaluate the power of ABSI for predicting the development of diabetes, hypertension, and dyslipidemia compared with BMI and WC in Japanese adults. This large retrospective cohort study is the first report evaluating ABSI in a Japanese population.

## Materials and Methods

### Study population

**Fig 1:**
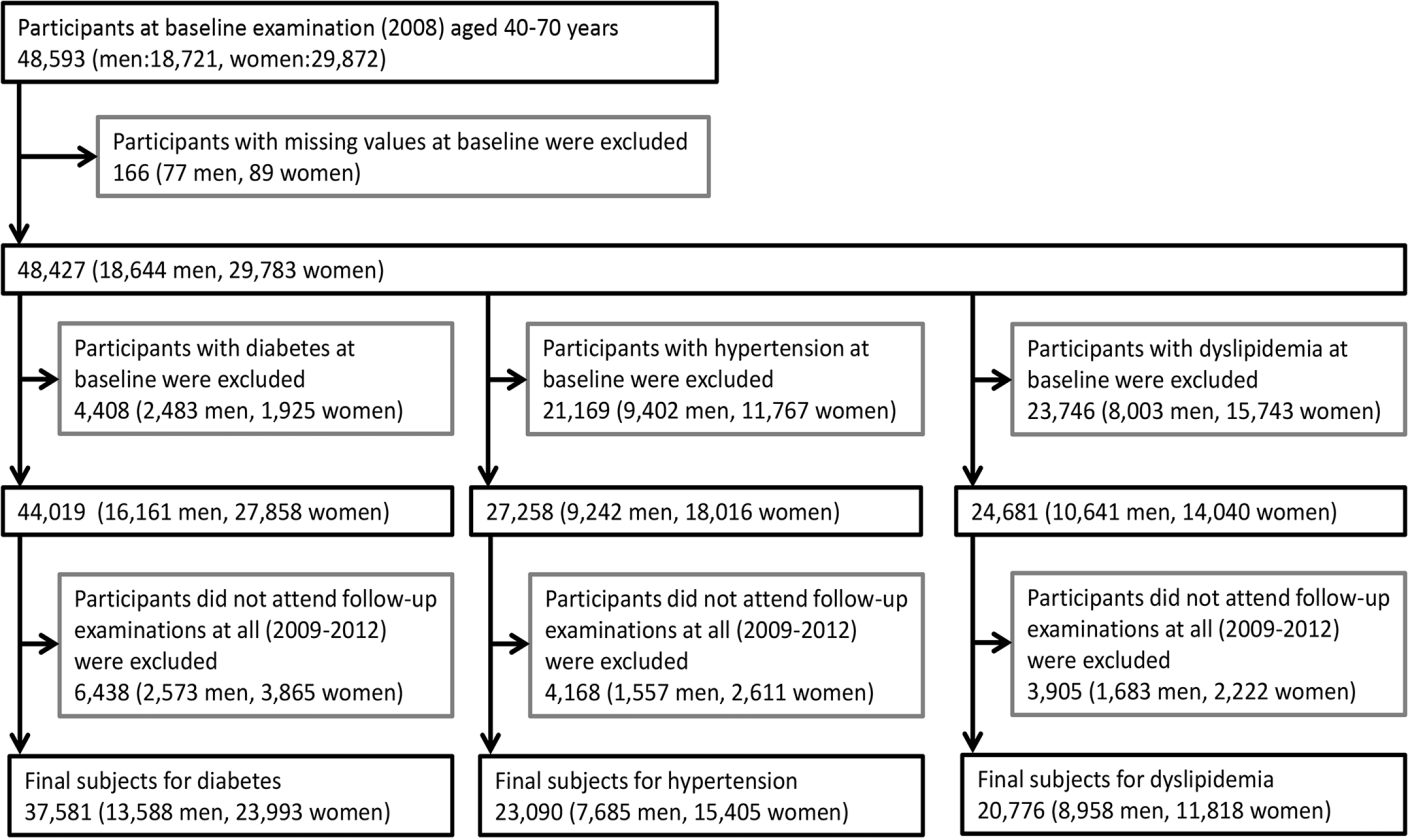
Selection of the subjects in this study for examining the incidence of diabetes, hypertension, and dyslipidemia.

We examined the predictive power of ABSI for development of diabetes, hypertension, and dyslipidemia by retrospective analysis of annual health examination data collected from members of Chiba City National Health Insurance for the period 2008 to 2012. The patient selection process is shown in Fig 1. In 2008, 48,593 members (18,721 men, 29,872 women) age 40−70 years attended annual health examinations at designated clinics or hospitals in the city. We excluded 166 participants (77 men, 89 women) with missing information at baseline. For the analysis of incidence of diabetes, hypertension, and dyslipidemia during follow-up, 4,408 participants (2,483 men, 1,925 women) with diabetes, 21,169 (9,402 men and 11,767 women) with hypertension, and 23,746 participants (8,003 men, 15,743 women) with dyslipidemia at baseline were excluded. Diabetes was defined as fasting plasma glucose ≥ 7.0 mmol/L, hemoglobin A1c (HbA1c) ≥ 6.5%, or current use of medications for diabetes as indicated by a self-report questionnaire in accordance with guidelines of the Japanese Diabetes Society [20]. Blood samples were drawn for measurement of plasma glucose during fasting and for measurement of HbA1c in the non-fasting period. Hypertension was defined as systolic blood pressure (SBP) ≥ 140 mmHg, diastolic blood pressure (DBP) ≥ 90 mmHg, or current use of antihypertensive medications as indicated by a self-report questionnaire according to the guidelines for the management of hypertension [21]. Dyslipidemia was defined as low-density lipoprotein cholesterol (LDL-C) ≥ 140 mg/dL, high-density lipoprotein cholesterol (HDL-C) < 40 mg/dL, or current use of medication for dyslipidemia according to the guidelines for prevention of arteriosclerosis [22]. Furthermore, participants who did not attend annual health examinations for the period 2009 to 2012 were excluded, comprising 6,438 for diabetes analysis (2,573 men, 3,865 women), 4,168 for hypertension analysis (1,557 men, 2,611 women), and 3,905 for dyslipidemia analysis (1,683 men, 2,222 women). The final subject populations for analysis of new disease incidence during follow-up were 37,581 confirmed non-diabetics (13,588 men, 23,993 women), 23,090 confirmed non-hypertensives (7,685 men, 15,405 women), and 20,776 without dyslipidemia (8,958 men, 11,818 women). Thus, we could not follow the subjects completely. There were 6,438 dropouts during follow-up in the baseline non-diabetic cohort (14.6% of the total), 4,168 (15.3%) in the baseline non-hypertensive cohort, and 3,905 (15.8%) in the baseline non-dyslipidemic cohort (S1 Table). There were marked differences in baseline characteristics between these dropouts and retained study subjects. For example, the dropouts were younger, more likely to be smokers, heavier, and had lower mean ABSI values compared with retained subjects in all three subgroups. In addition, dropouts had higher BMIs than the baseline non-diabetic and non-hypertensive subgroups.

### Ethics statement

Consent was not obtained from subjects because this study was performed using the data obtained from annual health examinations and conducted in accordance with the Act on Assurance of Medical Care for the Elderly enforced by the Ministry of Health, Labour and Welfare. To ensure anonymity, personal identifiers (e.g., name, address, and telephone number) were removed from the records, date of birth was converted into the 1st of each month, and personal ID was converted into a random number at Chiba City Hall prior to release for analysis. The Institutional Review Board of Chiba University Graduate School of Medicine approved this study. All procedures were in accordance with the Ethical Guidelines for Epidemiological Research of the Japanese Ministry of Education, Culture, Sports, Science and Technology and the Japanese Ministry of Health, Labour and Welfare. The study was conducted in accordance with the Declaration of Helsinki.

### Baseline examination

We defined the results from the 2008 annual health examinations as the baseline data. Height, weight, WC, SBP, and DBP were measured and blood samples drawn during periods of fasting or non-fasting. Weight and height were measured without shoes and while wearing light clothes. WC was measured at the level of the umbilicus with an anthropometric measuring tape. However, when the umbilicus level was drooping due to excess abdominal fat accumulation, WC was measured at the midpoint between the lower border of the rib cage and the iliac crest. BMI was calculated as weight in kilograms divided by the square of height in meters. ABSI was calculated as WC/(BMI^2/3^height^1/2^), with WC and height in meters [13]. Serum glucose, HbA1c, LDL-C, HDL-C, AST, ALT, and GGT were determined by commercial clinical laboratories. Subjects were categorized according to the following parameters. (1) Diabetes category (normal, borderline, and diabetic) was determined on the basis of baseline fasting plasma glucose, HbA1c, or self-reported use of medications for diabetes according to the guidelines of the Japanese Diabetes Society [20]; with normal (no diabetes) defined as fasting plasma glucose < 6.1 mmol/L or HbA1c < 6.0% and no history of diabetes medication use; borderline as fasting plasma glucose between 6.1 and < 7.0 mmol/L or HbA1c between 6.0% and < 6.5% and no use of medication for diabetes; and diabetic as fasting plasma glucose ≥ 7.0 mmol/L, or HbA1c ≥ 6.5% or current use of medication for diabetes. (2) Smoking status (non-smoker, smoker) was defined using a self-report questionnaire. Plasma concentrations of HDL-C, AST, ALT, and GGT were transformed logarithmically because the distributions of these variables were not normal.

### Incidence of diabetes, hypertension, and dyslipidemia

Results of annual health examinations from 2009 to 2012 were used as the follow-up data. Each follow-up examination included routine blood analysis, blood pressure measurement, and completion of a self-report questionnaire about medications for diabetes, hypertension, and dyslipidemia. Fasting plasma glucose (or HbA1c), LDL-C, and HDL-C were measured at each follow-up examination. Subjects with fasting plasma glucose ≥ 7.0 mmol/L, HbA1c ≥ 6.5%, or reporting medication for diabetes during follow-up but without diabetes at baseline were defined as new diabetic patients. Similarly, subjects with SBP ≥ 140 mmHg, DBP ≥ 90 mmHg, or reporting medication for hypertension during follow-up but without hypertension at baseline were defined as new hypertensive patients. Subjects with LDL-C ≥ 140 mg/dL, HDL-C < 40 mg/dL, or reporting medication for dyslipidemia but without dyslipidemia at baseline were defined as new dyslipidemic patients.

### Statistical analysis

Continuous variables are presented as mean ± standard deviation. For each disease, comparison of variables between cases and controls was performed using unpaired t-tests or chi-square tests as appropriate and in case-matched subgroups using McNemar’s test or the paired t-test as appropriate. We also calculated the standardized difference (SD) between cases and controls for all subjects and for case-matched subgroups [23]. The correlations among BMI, WC, and ABSI were evaluated by Pearson’s correlation coefficients.

#### Calculating Z-scores

**Fig 2:**
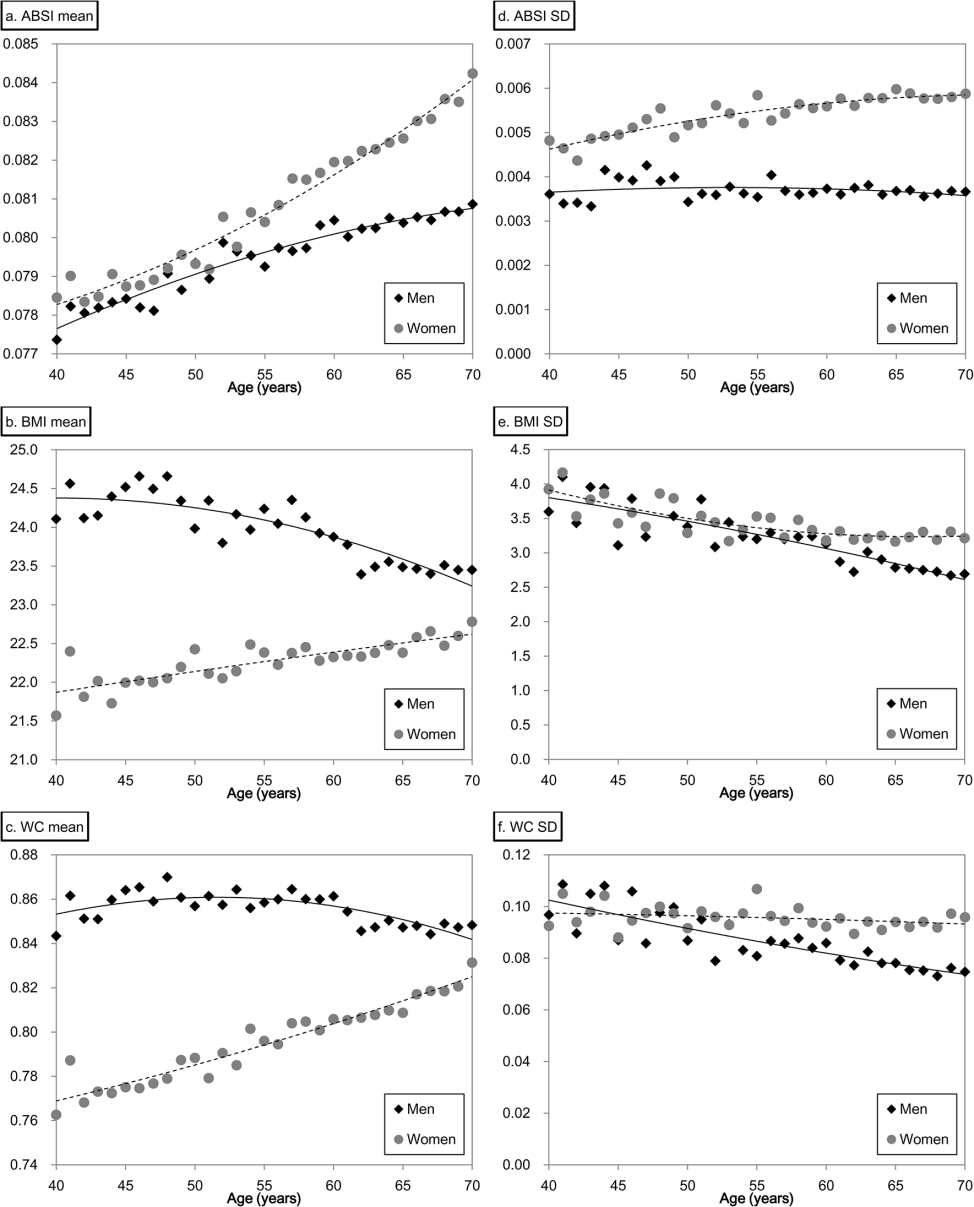
Mean and standard deviation of ABSI, BMI, and WC stratified by sex and age. Abbreviations: BMI, body mass index; WC, waist circumference; ABSI, a body shape index. Each marker shows mean and standard deviation stratified by sex and age. Black squares, men; gray circles, women.

For standardization, Z-scores for height, weight, BMI, WC, and ABSI were calculated as reported by Krakauer et al. [13]. First, we computed the sample mean and standard deviation for each age (in years) separately for all men and women (48,759 subjects in total) who did not have missing data at baseline (each marker in Fig 2). Second, we obtained the predicted mean and standard deviation for each age from the quadratic curve that described the change in mean or standard deviation with age (trend lines in Fig 2). From measurement values, predicted means, and standard deviations, Z-scores were calculated as follows: (measurement value—predicted mean)/predicted standard deviation.

#### Logistic regression analysis

We conducted logistic regression analysis to examine the associations of standardized BMI, WC, and ABSI (Z-scores) with incidences of diabetes, hypertension, and dyslipidemia. Logistic regression analyses were performed using two model types: sex- and age-adjusted models and multivariable-adjusted models including the covariates of sex, age, diabetes category (normal, borderline, or diabetes), SBP, DBP, LDL-C, log [HDL-C], log [AST], log [ALT], log [GGT], and smoking habit at baseline depending on application. All factors were included in the model for new diabetes incidence in the baseline non-diabetic cohort except for diabetes category; all except SBP and DBP for analysis of new hypertension incidence in the baseline non-hypertensive cohort; and all except LDL-C and log [HDL-C] for analysis of new dyslipidemia incidence in the baseline non-dyslipidemic cohort. Each regression was conducted both with and without BMI adjustment. For sensitivity analysis, logistic regression was also performed in subgroups stratified by sex. The model selection criteria were Akaike’s information criterion (AIC) and area under the curve (AUC) from receiver operating characteristic analysis.

#### Case matching

As shown in Table 1, there were significant differences in baseline parameters and a large patient number discrepancy between cases (subjects who would eventually be diagnosed) and controls (those who would not be diagnosed with diabetes, hypertension, or dyslipidemia during follow-up). Therefore, case selection was performed by employing the propensity score matching method with a greedy 5-to-1 digit-matching algorithm [24] for control of baseline characteristics. Variables used for calculating propensity scores were sex, age, diabetes category, smoking habit, SBP, DBP, LDL-C, log [HDL-C], log [aspartate aminotransferase, AST], log [alanine aminotransferase, ALT], and log [gamma-glutamyltransferase, GGT] depending on application. That is, all variables except diabetes category were included for the analysis of new diabetes incidence; all except SBP and DBP for analysis of new hypertension incidence; and all except LDL-C and log [HDL-C] for analysis of new dyslipidemia incidence. The greedy 5-to-1 digit-matching algorithm yields “best” matches first and then “next-best” matches in a hierarchical sequence until no more matches can be made. Greedy 5-to-1 digit-matching means that the cases were first matched to controls on 5 digits of the propensity score. For those that did not match, cases were then matched to controls on 4 digits of the propensity score. This continued down to a 1-digit match on propensity score for subjects that remained unmatched. This algorithm yields 1:1 case−control pairs [24]. After this process, there were 1,947 pairs for diabetes, 6,962 pairs for hypertension, and 7,263 pairs for dyslipidemia. Conditional logistic regression models were used to assess the associations of BMI, WC, and ABSI with each disease in case-matched subgroups yielded by the propensity score matching method.

**Table 1:** Baseline characteristics of all subjects divided according to the absence of disease at baseline (diabetes, hypertension, dyslipidemia) and subdivided according to onset of new disease during follow-up (cases vs. controls).

	Diabetes				Hypertension				Dyslipidemia			
	Without Incidence (controls)	With Incidence (cases)	p-value	SD (%)	Without Incidence (controls)	With Incidence (cases)	p-value	SD (%)	Without Incidence (controls)	With Incidence (cases)	p-value	SD (%)
Number of subjects	35,634	1,947			16,127	6,963			13,513	7,263		
%Men ^a^	35.3	52.6	<0.001	-21.8	31.1	38.3	<0.001	-8.8	44.6	40.4	<0.001	5.6
Age (years) ^b^	63.1 (6.7)	64.5 (5.4)	<0.001	-20.6	61.5 (7.7)	63.9 (5.8)	<0.001	-32.8	62.4 (7.6)	63.3 (6.5)	<0.001	-13.2
Height (cm) ^b^	158.1 (8.2)	159.5 (8.4)	<0.001	-17.6	158.2 (8.1)	158.2 (8.3)	0.596	-0.8	159.5 (8.4)	158.7 (8.2)	<0.001	10.4
Weight (kg) ^b^	56.8 (10.1)	61.5 (10.8)	<0.001	-46.6	55.0 (9.6)	57.1 (10.0)	<0.001	-21.9	56.8 (10.3)	57.5 (10.4)	<0.001	-6.5
Waist (cm) ^b^	81.8 (8.8)	85.9 (9.0)	<0.001	-46.9	79.8 (8.6)	82.2 (8.6)	<0.001	-27.2	80.6 (9.1)	82.1 (8.8)	<0.001	-16.4
BMI (kg/m^2^) ^b^	22.6 (3.0)	24.1 (3.4)	<0.001	-48.0	21.9 (2.8)	22.7 (2.9)	<0.001	-29.2	22.2 (3.1)	22.7 (3.1)	<0.001	-16.2
ABSI ^b^	0.0815 (0.00532)	0.0818 (0.00479)	0.008	-5.2	0.0813 (0.00544)	0.0816 (0.00525)	<0.001	-5.4	0.0810 (0.00523)	0.0815 (0.00515)	<0.001	-8.4
Diabetes category ^a^												
Normal	91.7	43.5	<0.001	156.9	87.6	83.0	<0.001	12.2	85.3	83.3	<0.001	5.1
Borderline	8.3	56.6		-54.5	7.6	9.3		-1.8	8.4	9.3		-0.9
Diabetes	-	-			4.9	7.7		-3.6	6.3	7.4		-1.2
SBP ^b^	128 (16.7)	133 (16.4)	<0.001	-28.4	117 (11.7)	126 (8.7)	<0.001	-82.2	128 (17.2)	129 (16.8)	<0.001	-6.5
DBP ^b^	77 (10.6)	79 (10.4)	<0.001	-17.9	71 (8.5)	76 (7.8)	<0.001	-58.7	76 (10.9)	77 (10.7)	0.006	-4.0
LDL-C ^b^	128 (30.3)	128 (32.1)	0.857	0.4	128 (30.4)	129 (30.9)	0.121	-2.2	106 (19.2)	122 (15.1)	<0.001	-89.3
Log HDL-C ^b^	4.15 (0.26)	4.06 (0.26)	<0.001	34.4	4.17 (0.26)	4.14 (0.27)	<0.001	13.4	4.21 (0.24)	4.12 (0.25)	<0.001	37.4
Log AST ^b^	3.12 (0.28)	3.19 (0.35)	<0.001	-25.7	3.10 (0.28)	3.13 (0.30)	<0.001	-11.6	3.13 (0.31)	3.12 (0.30)	0.026	3.2
Log ALT ^b^	2.95 (0.43)	3.15 (0.50)	<0.001	-46.2	2.91 (0.42)	2.98 (0.45)	<0.001	-14.9	2.93 (0.44)	2.95 (0.45)	<0.001	-5.5
Log GGT ^b^	3.24 (0.64)	3.54 (0.71)	<0.001	-47.5	3.14 (0.60)	3.28 (0.65)	<0.001	-23.1	3.27 (0.70)	3.28 (0.68)	0.959	-0.1
Smoking habit ^a^	13.2	19.3	<0.001	-6.6	13.8	13.9	0.803	-0.14	16.7	13.7	<0.001	3.2

ABSI, a body shape index; WC, waist circumference; BMI, body mass index; SD, standardized difference; SBP, systolic blood pressure; DBP, diastolic blood pressure; LDL-C, low-density lipoprotein cholesterol; HDL-C, high-density lipoprotein cholesterol; AST, aspartate aminotransferase; ALT, alanine aminotransferase; GGT, gamma-glutamyltransferase

^a^ Percentage and p-value by the chi-square test

^b^ Mean (standard deviation) and p-value by the unpaired t-test

All comparisons were planned and all tests two-tailed. A p-value < 0.05 was considered statistically significant. All statistical analyses were performed using SAS software version 9.4 (SAS Institute, Cary, NC) and STATA13 software package (Stata Corp., College Station, TX).

## Results

The mean ABSI for the entire cohort increased with age in both men and women (Fig 2A). Means were similar in younger men and women (< 55 years) but the increase with age was larger in women thereafter and ABSI was more variable in women at all ages (Fig 2D). In contrast, mean BMI (Fig 2B) and WC (Fig 2C) exhibited modest age-dependent decreases in men (larger for BMI), while women exhibited age-dependent increases with similar trajectories. The SDs of BMI and WC were similar in men and women, with a nearly parallel age-dependent decrease (Fig 2E and 2F). The standardized WC was strongly correlated with weight (r = 0.803) and with BMI (r = 0.809), while ABSI did not correlate with weight (r = −0.006) or BMI (r = −0.034) and the correlation with WC was only moderate (r = 0.530).

The baseline characteristics of all subjects (data from 2008), with and without eventual development of the disease of interest during follow-up (2009−2012), are shown in Table 1. Almost all baseline characteristics differed between the case group and control group. In total, 1,947 of 37,581 subjects confirmed to be non-diabetic at baseline were eventually diagnosed with diabetes during the follow-up period (5.2%); 6,963 of 23,090 subjects confirmed to be non-hypertensive at baseline were eventually diagnosed with hypertension (30.2%); and 7,263 of 20,776 subjects without dyslipidemia at baseline were eventually diagnosed with dyslipidemia (35.0%). For all three diseases, BMI, WC, and ABSI at baseline were significantly larger in the case group than the control group (p < 0.001).

### Logistic regression analyses

Table 2 shows the odds ratios (ORs) of standardized BMI, WC, and ABSI values (Z-scores) for development of diabetes, hypertension, and dyslipidemia in age- and sex-adjusted models.

**Table 2:** Associations between disease incidence (diabetes, hypertension, dyslipidemia) and Z-scores for BMI, WC, and ABSI in sex- and age-adjusted models.

	OR (95% CI)	p-value for OR	AIC	AUC (95% CI)	p-value for AUC^1^
Diabetes					
Without BMI adjustment^2^					
BMI	1.47 (1.41−1.54)	<0.001	14733	0.6648 (0.6531−0.6764)	<0.001
WC	1.46 (1.40−1.53)	<0.001	14763	0.6605 (0.6489−0.6721)	<0.001
ABSI	1.11 (1.06−1.16)	<0.001	15003	0.6176 (0.6053−0.6299)	reference
With BMI adjustment^3^					
WC	1.18 (1.09−1.27)	<0.001	14718	0.6669 (0.6553−0.6785)	0.039
ABSI	1.14 (1.09−1.19)	<0.001	14706	0.6680 (0.6564−0.6796)	reference
Hypertension					
Without BMI adjustment^2^					
BMI	1.36 (1.32−1.40)	<0.001	27277	0.6237 (0.6161−0.6313)	<0.001
WC	1.27 (1.23−1.31)	<0.001	27394	0.6146 (0.6069−0.6223)	<0.001
ABSI	1.02 (0.99−1.05)	0.188	27630	0.5943 (0.5866−0.6020)	reference
With BMI adjustment^3^					
WC	1.03 (0.98−1.08)	0.273	27278	0.6237 (0.6161−0.6313)	0.383
ABSI	1.03 (0.99−1.06)	0.079	27276	0.6238 (0.6162−0.6314)	reference
Dyslipidemia					
Without BMI adjustment^2^					
BMI	1.20 (1.16−1.24)	<0.001	26619	0.5721 (0.5641−0.5802)	<0.001
WC	1.19 (1.16−1.23)	<0.001	26623	0.5706 (0.5625−0.5786)	<0.001
ABSI	1.05 (1.02−1.08)	0.001	26756	0.5504 (0.5422−0.5585)	reference
With BMI adjustment^3^					
WC	1.10 (1.05−1.15)	<0.001	26607	0.5734 (0.5653−0.5814)	0.482
ABSI	1.05 (1.02−1.08)	<0.001	26608	0.5736 (0.5655−0.5816)	reference

OR, odds ratio; CI, confidence interval; ABSI, a body shape index; WC, waist circumference; BMI, body mass index; AIC, Akaike’s information criterion; AUC, area under the curve

^1^ p-value with Bonferroni’s correction

^2^ Adjusted for sex and age (continuous variable, year)

^3^ Adjusted for BMI Z-score and the variables described above

For diabetes without BMI adjustment, all three parameters (BMI, WC, and ABSI) were positively associated with disease incidence (p < 0.001). However, the larger AIC and significantly smaller AUC in the model with ABSI compared with models with BMI (p < 0.001) or WC (p < 0.001) indicate that the predictive power of ABSI is weaker than those of BMI or WC for diabetes. With BMI adjustment, the predictive powers of WC and ABSI remained significant (both p < 0.001). The result of smaller AIC and significantly greater AUC in the model with ABSI than that with WC (p = 0.039) suggests that the predictive power of ABSI independent of BMI for diabetes is greater than that of WC. However, the observation that AUCs was not significantly different (p = 0.180) with ABSI and WC in the multivariable-adjusted models (Table 3) indicates that both parameters have similar predictive powers.

**Table 3:** Associations between new disease incidence (diabetes, hypertension, and dyslipidemia) and Z-scores for BMI, WC, and ABSI as revealed by logistic regression analysis of all subjects in the multivariable-adjusted model.

	OR (95% CI)	p-value for OR	AIC	AUC (95% CI)	p-value for AUC^1^
Diabetes					
Without BMI adjustment^2^					
BMI	1.26 (1.20−1.32)	<0.001	14426	0.6983 (0.6970−0.7097)	<0.001
WC	1.24 (1.18−1.31)	<0.001	14438	0.6971 (0.6857−0.7085)	<0.001
ABSI	1.06 (1.01−1.11)	0.015	14503	0.6903 (0.6788−0.7017)	reference
With BMI adjustment^3^					
WC	1.09 (1.01−1.18)	0.025	14423	0.6988 (0.6874−0.7102)	0.180
ABSI	1.09 (1.04−1.14)	0.001	14416	0.6992 (0.6878−0.7106)	reference
Hypertension					
Without BMI adjustment^2^					
BMI	1.32 (1.27−1.37)	<0.001	27160	0.6321 (0.6245−0.6397)	<0.001
WC	1.22 (1.18−1.26)	<0.001	27264	0.6244 (0.6167−0.6320)	<0.001
ABSI	1.00 (0.97−1.02)	0.742	27401	0.6147 (0.6071−0.6224)	reference
With BMI adjustment^3^					
WC	1.01 (0.96−1.06)	0.820	27162	0.6320 (0.6244−0.6397)	0.930
ABSI	1.01 (0.98−1.04)	0.440	27161	0.6321 (0.6245−0.6397)	reference
Dyslipidemia					
Without BMI adjustment^2^					
BMI	1.15 (1.12−1.19)	<0.001	26556	0.5796 (0.5715−0.5876)	<0.001
WC	1.15 (1.11−1.19)	<0.001	26556	0.5791 (0.5710−0.5872)	<0.001
ABSI	1.04 (1.01−1.07)	0.011	26626	0.5696 (0.5615−0.5777)	reference
With BMI adjustment^3^					
WC	1.08 (1.03−1.14)	0.002	26548	0.5805 (0.5725−0.5586)	0.597
ABSI	1.05 (1.02−1.08)	0.002	26548	0.5807 (0.5726−0.5887)	reference

OR, odds ratio; CI, confidence interval; ABSI, a body shape index; WC, waist circumference; BMI, body mass index; AIC, Akaike’s information criterion; AUC, area under the curve

^1^ p-value with Bonferroni’s correction

^2^ Adjusted for sex and all baseline characteristics, including age (continuous variable, year), diabetes category, systolic blood pressure, diastolic blood pressure, low-density lipoprotein cholesterol, log [high-density lipoprotein cholesterol], log [aspartate aminotransferase], log [alanine aminotransferase], log [gamma-glutamyltransferase] and smoking habit, depending on application. The diabetes category was omitted for the analysis of new diabetes incidence, systolic blood pressure and diastolic blood pressure were omitted for analysis of new hypertension incidence, and low-density lipoprotein cholesterol and log [high-density lipoprotein cholesterol] were omitted for the analysis of new dyslipidemia incidence.

^3^ Adjusted for BMI Z-score and the variables described above

For hypertension without BMI adjustment, elevated BMI and WC were significantly associated with increased disease incidence (p < 0.001), while ABSI was not (p = 0.188), as shown in Table 2. The greater AIC was observed in the model with ABSI than those with BMI and WC, while a significantly smaller AUC was observed in the ABSI model compared with those with BMI and WC (both p < 0.001). With BMI adjustment, neither WC nor ABSI was significantly associated with the incidence of hypertension (p = 0.273 and p = 0.079, respectively). The AICs were similar in models with WC or ABSI, and the AUCs did not differ significantly (p = 0.383). Thus, similar to diabetes, the predictive power of ABSI for hypertension incidence was weaker than those of BMI and WC when BMI was not adjusted, while the powers of ABSI and WC were similar after BMI adjustment. These results are consistent with those in the multivariable-adjusted models (Table 3), where BMI and WC were significant predictors of hypertension incidence (p < 0.001), while ABSI was not (p = 0.742). With BMI adjustment, neither WC nor ABSI was a significant predictor of hypertension.

**Fig 3:**
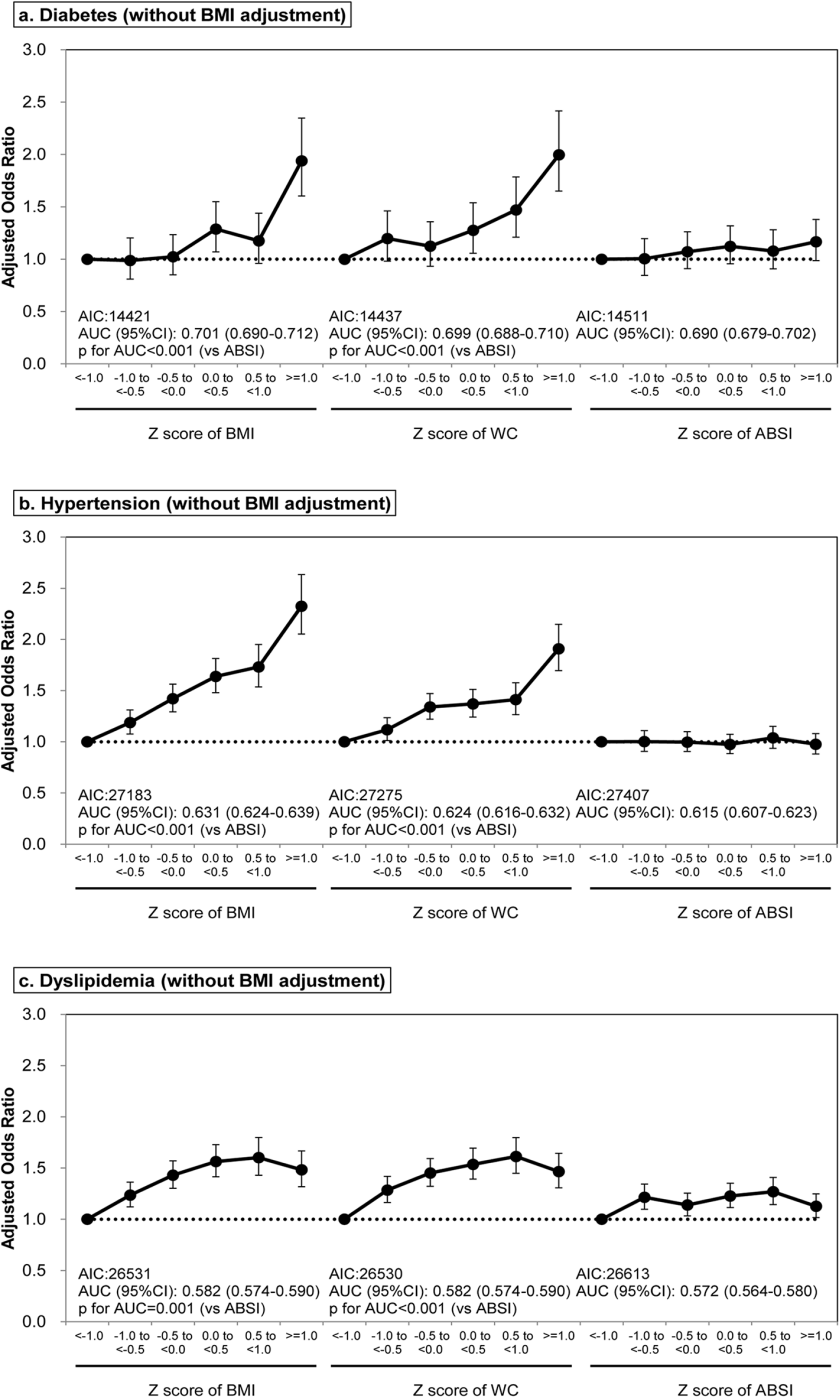
Odds ratio and 95% confidence interval of BMI, WC, and ABSI for predicting the onset of diabetes, hypertension, and dyslipidemia without BMI adjustment. Abbreviations: BMI, body mass index; WC, waist circumference; ABSI, a body shape index; AIC, Akaike’s information criterion; AUC, area under the curve; 95% CI, 95% confidence interval. Models were adjusted for sex and baseline characteristics, including age (continuous variable, year), diabetes category, systolic blood pressure, diastolic blood pressure, low-density lipoprotein cholesterol, log [high-density lipoprotein cholesterol], log [aspartate aminotransferase], log [alanine aminotransferase], log [gamma-glutamyltransferase] and smoking habit, depending on application. Diabetes category was excluded for the analysis of diabetes, systolic blood pressure and diastolic blood pressure for the analysis of hypertension, and low-density lipoprotein cholesterol and log [high-density lipoprotein cholesterol] for the analysis of dyslipidemia.

**Fig 4:**
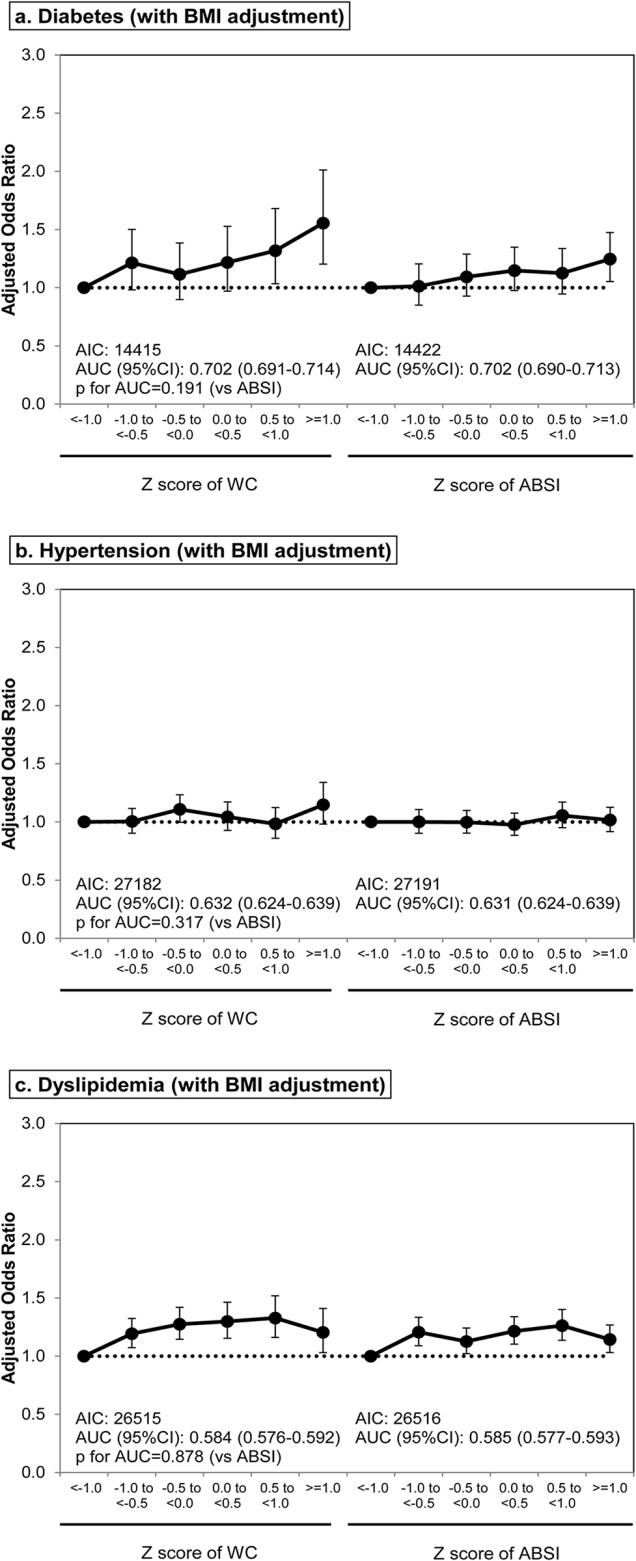
Odds ratio and 95% confidence interval of WC and ABSI categories for development of diabetes, hypertension and dyslipidemia with BMI adjustment. Abbreviations: BMI, body mass index; WC, waist circumference; ABSI, a body shape index; AIC, Akaike’s information criterion; AUC, area under the curve; 95% CI, 95% confidence interval. Adjusted for BMI category in addition to same variables as shown in Fig 3.

For dyslipidemia without BMI adjustment, all three parameters (BMI, WC, and ABSI) were positively associated with disease incidence (p < 0.001, p < 0.001, and p = 0.001, respectively) in sex- and age-adjusted models. Again, the greater AIC and significantly smaller AUC was observed in the model with ABSI compared with those with BMI or WC (both p < 0.001). After BMI adjustment, WC and ABSI were significantly associated with the incidence of dyslipidemia (both p < 0.001), showing similar AICs and AUCs (p = 0.482). These results are consistent with those for multivariable-adjusted models (Table 3), where all three parameters were predictive of dyslipidemia without BMI adjustment (p < 0.001, p < 0.001, and p = 0.011, respectively) but the predictive power of ABSI was slightly weaker as indicated by the AIC and AUC values. Both WC and ABSI were also significant predictors in models with BMI adjustment (both p = 0.002). The ORs were also calculated and data were stratified by BMI, WC, and ABSI Z-score ranges both without (Fig 3) and with BMI correction (Fig 4). Results were consistent with analysis of the continuous data (Table 3).

### Sensitivity analysis

We also performed logistic regression analysis in subgroups stratified by sex (Table 4). These results were generally consistent with those for the individual baseline non-diabetic, non-hypertensive, and non-dyslipidemic groups (Table 3). However, in men, compared with ABSI, BMI and WC were not stronger predictors of diabetes when BMI was not adjusted (Table 4) (p for AUC = 0.999 and 0.829, respectively). Furthermore, the interactions between sex and BMI and between sex and WC were significant (p < 0.001), while that between sex and ABSI was not (p = 0.465), suggesting that the effects of high BMI and WC on diabetes incidence are greater in women than in men, but the effect of ABSI does not differ by sex.

**Table 4:** Sensitivity analysis in subgroups stratified by sex.

	Men					Women					
	OR (95% CI)	p-value for OR	AIC	AUC (95% CI)	p−value for AUC^1^	OR (95% CI)	p−value for OR	AIC	AUC (95% CI)	p−value for AUC^1^	p-value for interaction^2^
Diabetes											
Without BMI adjustment^3^											
BMI	1.12 (1.04−1.21)	0.002	7057	0.6385 (0.6212−0.6557)	0.999	1.39 (1.30−1.48)	<0.001	7350	0.7039 (0.6872−0.7206)	<0.001	<0.001
WC	1.11 (1.03−1.20)	0.005	7059	0.6384 (0.6211−0.6556)	0.829	1.39 (1.29−1.49)	<0.001	7358	0.7016 (0.6848−0.7183)	<0.001	<0.001
ABSI	1.05 (0.98−1.12)	0.132	7065	0.6369 (0.6197−0.6542)	reference	1.08 (1.01−1.15)	0.032	7437	0.6891 (0.6724−0.7057)	reference	0.465
With BMI adjustment^4^											
WC	1.04 (0.92−1.17)	0.566	7059	0.6386 (0.6213−0.6556)	0.227	1.17 (1.06−1.30)	0.002	7343	0.7048 (0.6881−0.7215)	0.789	<0.001
ABSI	1.07 (0.99−1.14)	0.053	7056	0.6396 (0.6224−0.6569)	reference	1.11 (1.04−1.19)	0.003	7343	0.7049 (0.6882−0.7216)	reference	0.607
Hypertension											
Without BMI adjustment^3^											
BMI	1.26 (1.19−1.34)	<0.001	9632	0.6157 (0.6028−0.6287)	0.001	1.36 (1.30−1.43)	<0.001	17487	0.6355 (0.6260−0.6450)	<0.001	0.061
WC	1.20 (1.13−1.27)	<0.001	9654	0.6116 (0.5986−0.6246)	0.016	1.23 (1.18−1.29)	<0.001	17574	0.6254 (0.6159−0.6350)	<0.001	0.422
ABSI	1.00 (0.95−1.05)	0.978	9693	0.6039 (0.5909−0.6170)	reference	0.99 (0.96−1.03)	0.619	17674	0.6157 (0.6062−0.6253)	reference	0.637
With BMI adjustment^4^											
WC	1.01 (0.92−1.10)	0.903	9634	0.6157 (0.6028−0.6287)	0.665	1.01 (0.95−1.07)	0.817	17489	0.6354 (0.6259−0.6449)	0.395	0.166
ABSI	1.02 (0.97−1.07)	0.418	9633	0.6159 (0.6029−0.6288)	reference	1.00 (0.97−1.04)	0.809	17489	0.6354 (0.5259−0.6449)	reference	0.373
Dyslipidemia											
Without BMI adjustment^3^											
BMI	1.19 (1.13−1.24)	<0.001	11208	0.5748 (0.5623−0.5873)	0.004	1.14 (1.09−1.19)	<0.001	15317	0.5775 (0.5670−0.5880)	0.002	0.105
WC	1.17 (1.12−1.23)	<0.001	11213	0.5735 (0.5611−0.5860)	0.001	1.14 (1.10−1.19)	<0.001	15314	0.5783 (0.5678−0.5888)	<0.001	0.222
ABSI	1.04 (0.99−1.08)	0.115	11252	0.5621 (0.5495−0.8747)	reference	1.04 (1.00−1.79)	0.040	15351	0.5688 (0.5582−0.5793)	reference	0.965
With BMI adjustment^4^											
WC	1.07 (0.99−1.16)	0.117	11208	0.5755 (0.5630−0.5879)	0.171	1.09 (1.03−1.16)	0.006	15312	0.5794 (0.5689−0.5899)	0.186	0.290
ABSI	1.05 (1.00−1.99)	0.030	11205	0.5762 (0.5637−0.5886)	reference	1.05 (1.01−1.08)	0.020	15314	0.5790 (0.5685−0.5895)	reference	0.821

OR, odds ratio; CI, confidence interval; ABSI, a body shape index; WC, waist circumference; BMI, body mass index; AIC, Akaike’s information criterion; AUC, area under the curve

Subjects were 13,588 men and 23,993 women without diabetes at baseline, 7,685 men and 15,405 women without hypertension at baseline, and 8,958 men and 11,818 women without dyslipidemia at baseline.

^1^ p-value with Bonferroni’s correction

^2^ p-value for interaction of sex with BMI, WC, and ABSI

^3^Adjusted for baseline characteristics, including age (continuous variable, year), diabetes category, systolic blood pressure, diastolic blood pressure, low-density lipoprotein cholesterol, log [high-density lipoprotein cholesterol], log [aspartate aminotransferase], log [alanine aminotransferase], log [gamma-glutamyltransferase] and smoking habit, except for diabetes category in the analysis of diabetes, systolic blood pressure and diastolic blood pressure in the analysis of hypertension, and low-density lipoprotein cholesterol and log [high-density lipoprotein cholesterol] in the analysis of dyslipidemia.

^4^Adjusted for BMI Z-score and the variables described above

### Study of case-matched subset by propensity score

All baseline characteristics differed significantly between cases and controls for all three diseases in the total cohorts (Table 1), but no significant differences remained in the variables used for calculating propensity score following propensity score matching (Table 5), and all imbalances between cases and controls were less than 20% [23]. Table 6 shows the results of conditional logistic regression analysis for these case-matched subgroups. Results indicate that compared with WC and BMI, ABSI had weaker predictive power for the incidence of all three diseases.

**Table 5:** Baseline characteristics of controls and cases after propensity score matching.

	Diabetes				Hypertension				Dyslipidemia			
	Without Incidence (controls)	With Incidence (cases)	p-value	SD (%)	Without Incidence (controls)	With Incidence (cases)	p-value	SD (%)	Without Incidence (controls)	With Incidence (cases)	p-value	SD (%)
Number of subjects	1,947	1,947			6,962	6,962			7,263	7,263		
Men percent ^a^	53.6	52.6	0.477	1.4^c^	38.0	38.3	0.220	-0.4^c^	40.4	40.4	0.971	0.0^c^
Age (years)^b^	64.4 (5.6)	64.5 (5.4)	0.526	2.0^c^	63.9 (5.7)	63.9 (5.8)	0.382	0.9^c^	63.3 (6.8)	63.3 (6.5)	0.726	0.5^c^
BMI	23.5 (3.2)	24.1 (3.4)	<0.001	-19.3	22.1 (2.8)	22.7 (2.9)	<0.001	-20.2	22.3 (3.1)	22.7 (3.1)	<0.001	-12.3
WC	84.4 (8.5)	85.9 (9.0)	<0.001	-17.1	80.8 (8.5)	82.2 (8.6)	<0.001	-15.6	80.9 (9.0)	82.1 (8.8)	<0.001	-13.2
ABSI	0.0817 (0.0048)	0.0818 (0.0048)	0.217	-4.0	0.0816 (0.0053)	0.0816 (0.0052)	0.713	0.6	0.0812 (0.0052)	0.0815 (0.0051)	0.002	-5.0
Diabetes category ^a^												
Normal	89.3	43.5	<0.001	90.5	83.4	83.1	0.817	0.8^c^	83.2	83.3	0.235	-0.2^c^
Borderline	10.7	56.6		-64.4	9.3	9.3		-0.1^c^	9.0	9.3		-0.2^c^
Diabetes	-	-			7.4	7.6		-0.3^c^	7.7	7.4		0.3^c^
SBP (mmHg)	134 (17)	133 (16)	0.490	2.1^c^	118 (11)	126 (9)	<0.001	-78.2	129 (17)	129 (17)	0.958	-0.1^c^
DBP (mmHg)	79 (11)	79 (10)	0.983	-0.1^c^	71 (8)	76 (8)	<0.001	-56.6	77 (11)	77 (11)	0.944	0.1^c^
LDL-C (mg/dl)	128 (32)	128 (32)	0.664	1.4^c^	129 (31)	129 (31)	0.498	1.1^c^	107 (19)	122 (15)	<0.001	-88.0
Log HDL-C	4.06 (0.26)	4.06 (0.26)	0.988	0.0^c^	4.14 (0.26)	4.14 (0.27)	0.545	0.9^c^	4.21 (0.24)	4.12 (0.25)	<0.001	36.5
Log AST	3.18 (0.34)	3.19 (0.35)	0.307	-3.0^c^	3.13 (0.29)	3.13 (0.30)	0.788	-0.4^c^	3.12 (0.30)	3.12 (0.30)	0.463	1.2^c^
Log ALT	3.14 (0.48)	3.15 (0.50)	0.357	-2.3^c^	2.97 (0.44)	2.98 (0.45)	0.756	-0.5^c^	2.95 (0.43)	2.95 (0.45)	0.721	0.6^c^
Log GGT	3.55 (0.72)	3.54 (0.71)	0.936	0.2^c^	3.27 (0.63)	3.28 (0.65)	0.503	-0.9^c^	3.28 (0.69)	3.28 (0.68)	0.868	0.3^c^
Smoking habit ^a^	19.0	19.3	0.798	-0.3^c^	14.0	13.9	0.843	0.1^c^	14.0	13.7	0.701	0.2^c^

SD, standardized difference; SBP, systolic blood pressure; DBP, diastolic blood pressure; LDL-C, low-density lipoprotein cholesterol; HDL-C, high-density lipoprotein cholesterol; AST, aspartate aminotransferase; ALT, alanine aminotransferase; GGT, gamma-glutamyltransferase

^a^ Percentage and p-value by McNemar’s test

^b^ Mean (standard deviation) and p-value by paired t-test

^c^ These variables were used for calculating the propensity score

**Table 6:** Associations between disease incidence (diabetes, hypertension, and dyslipidemia) and Z-scores for BMI, WC, and ABSI as revealed by conditional logistic regression analysis of case-matched subsets obtained by propensity score matching.

	OR (95% CI)	p-value for OR	AIC	AUC (95% CI)	p-value for AUC^1^
Diabetes					
Without BMI adjustment					
BMI	1.20 (1.13−1.28)	<0.001	2666	0.5549 (0.5369−0.5729)	0.002
WC	1.18 (1.11−1.26)	<0.001	2676	0.5456 (0.5275−0.5636)	<0.001
ABSI	1.03 (0.96−1.10)	0.385	2700	0.5098 (0.4917−0.5280)	reference
With BMI adjustment^2^					
WC	1.02 (0.91−1.15)	0.680	2668	0.5546 (0.5366−0.5726)	0.682
ABSI	1.05 (0.98−1.12)	0.134	2666	0.5554 (0.5374−0.5734)	reference
Hypertension					
Without BMI adjustment					
BMI	1.26 (1.21−1.31)	<0.001	9512	0.5567 (0.5472−0.5662)	<0.001
WC	1.18 (1.14−1.23)	<0.001	9570	0.5419 (0.5323−0.5514)	<0.001
ABSI	0.99 (0.96−1.03)	0.713	9653	0.5025 (0.4929−0.5121)	reference
With BMI adjustment^2^					
WC	0.99 (0.94−1.05)	0.830	9514	0.5567 (0.5472−0.5663)	0.500
ABSI	1.00 (0.97−1.03)	0.995	9514	0.5567 (0.5472−0.5662)	reference
Dyslipidemia					
Without BMI adjustment					
BMI	1.14 (1.10−1.18)	<0.001	10015	0.5386 (0.5293−0.5480)	0.001
WC	1.15 (1.11−1.19)	<0.001	10006	0.5391 (0.5298−0.5485)	<0.001
ABSI	1.05 (1.02−1.09)	0.003	10062	0.5150 (0.5056−0.5244)	reference
With BMI adjustment^2^					
WC	1.11 (1.04−1.17)	0.001	10005	0.5409 (0.5315−0.5503)	0.600
ABSI	1.06 (1.02−1.09)	0.001	10006	0.5412 (0.5318−0.5506)	reference

OR, odds ratio; CI, confidence interval; ABSI, a body shape index; WC, waist circumference; BMI, body mass index; AIC, Akaike’s information criterion; AUC, area under the curve

^1^ p-value with Bonferroni’s correction

^2^ Adjusted for BMI

## Discussion

Our retrospective cohort study revealed that compared with BMI and WC, ABSI was not a better predictor of diabetes, hypertension, or dyslipidemia incidence in Japanese adults. In addition, using propensity score matching as an alternative evaluation method, the predictive power of ABSI for all three diseases was also lower than those of BMI and WC.

In American Caucasians, high ABSI accounted for a greater proportion of the total mortality hazard than did elevated BMI [13]. There are two possible reasons for this discrepancy. One is the different outcome measures used: all-cause mortality versus specific disease conditions. The other is racial differences. In regard to the first reason, a U-shaped or J-shaped association between all-cause mortality and BMI has been reported [1, 10, 13], while the associations of diabetes, hypertension, and dyslipidemia incidence [5] and prevalence [25, 26] with BMI are typically linear or nearly linear. In our study, near-linear associations between BMI and the incidence of diabetes and hypertension were also observed. In regard to the second reason, morphometric and physiological racial differences may alter the health implications of these indices. For example, the prevalence of obesity in Japanese adults was much lower than Americans [27]. In addition, East Asians, including the Japanese, are known to have limited innate capacity for insulin secretion, resulting in greater susceptibility to type 2 diabetes than Caucasians [28]. A prospective cohort study of Chinese adults of a smaller scale (n = 687) with the incidence of diabetes as the primary outcome has reported that the predictive power of ABSI was no better than that of WC or BMI [18]. In another study, the association of ABSI with hypertension incidence was found to be weaker than WC or BMI among 8,255 Indonesians [19]. Our results were consistent with those of these two studies of Asian subjects that examined the incidence of diabetes or hypertension as outcomes, even though the precise reasons for the discrepancy were unable to be ascertained. It should be noted that correlations between BMI, WC, and ABSI in our study were similar to those of Krakauer et al [13]. In brief, both studies found a high correlation between BMI and WC, a moderate correlation between ABSI and WC, but almost no correlation between ABSI and BMI.

ABSI was significantly associated with the incidence of diabetes and dyslipidemia even after adjustment for BMI. These BMI-independent associations may reflect the fact that ABSI represents central body mass, such as higher visceral fat accumulation, higher trunk fat accumulation, and lesser limb lean mass. Furthermore, in the sex- and age-adjusted models, the finding that the predictive power of ABSI for diabetes independent of BMI was significantly greater than that of WC, could be interesting (Table 2). If this is true, ABSI has an advantage over WC in predicting diabetes. However, this association was not observed in the multivariable-adjusted models (Table 3), after propensity score matching (Table 6), or in sensitivity analyses (Table 4). Thus, the stronger predictive power of ABSI might be confounded by other co-varying baseline characteristics.

We found that the predictive powers of BMI and WC for diabetes incidence are greater in women than in men, while that of ABSI does not differ by sex (Table 4). Cross-sectional studies in the Japanese population [29] and in the Framingham Heart Study [30] have shown that associations of metabolic risk factors, such as systolic and diastolic blood pressure, fasting glucose, and total cholesterol, with increasing volumes both of subcutaneous and visceral fat, strictly measured by computed tomography, were stronger in women than in men. Our observation of a sex difference in BMI and WC could be the same phenomenon, but this is not the case with ABSI. Thus, ABSI might have an implication for disease incidence that is different from BMI or WC. Further studies are necessary to conclude whether this difference is true or not.

The following four limitations in our study are conceivable. First, due to the retrospective cohort design, we could not obtain other potentially relevant data, such as alcohol consumption and levels of physical activity, which may confound the associations of BMI, WC, and ABSI with disease incidence. To correct this, we have made adjustment for potential confounding factors as much as possible using logistic regression analysis and the propensity score matching method. Second, fasting blood glucose data for some subjects were not available. To compensate for this, we employed HbA1c data and history of diabetes medication to obtain an accurate diagnosis. Third, some bias might exist that resulted in underestimation of associations because the reasons and outcomes of non-attendance of annual health checkup are unknown. Fourth, the generalizability of our findings might be limited because the subjects are mainly elderly adults living in one urban area of Japan with a relatively higher incidence of hypertension (30.2%) and dyslipidemia (35.0%) during the 4-year period. On the other hand, the major advantage of our study is that we obtained consistent results using two different covariate adjustment methods (logistic regression analysis and the propensity score matching) and also from sensitivity analyses. Furthermore, new-onset diabetes, hypertension, and dyslipidemia was confirmed both by self-reported medication use and physiological measures. Since subjects are not always aware of their disease, we may underestimate disease incidence when it is determined only by self-reported medication use.

In conclusion, ABSI was not a better predictor of diabetes, hypertension, and dyslipidemia incidence than BMI and WC in Japanese adults. However, ABSI had significant associations with the incidence of diabetes and dyslipidemia independently of BMI.

## Supporting Information

S1 Table Baseline characteristics of retained subjects and dropouts during follow-up. (DOCX)
