# Supplementary material for: Predictive Power of a Body Shape Index for Development of Diabetes, Hypertension, and Dyslipidemia in Japanese Adults: A Retrospective Cohort Study
Source: PLoS One. 2015 Jun 1;10(6):e0128972. doi: 10.1371/journal.pone.0128972 (PMC4451769; doi:10.1371/journal.pone.0128972)
Supplement: S1 Table — (DOCX) [file pone.0128972.s001.docx]

S1 Table. Baseline characteristics of retained subjects and dropouts during follow-up

|  | Diabetes | | | Hypertension | | | Dyslipidemia | | |
| --- | --- | --- | --- | --- | --- | --- | --- | --- | --- |
|  | Retained | Dropouts | p-value | Retained | Dropouts | p-value | Retained | Dropouts | p-value |
| Number of subjects | 37,581 | 6,438 |  | 23,090 | 4,168 |  | 20,776 | 3,905 |  |
| %Men ^a^ | 36.2 | 40.0 | <0.001 | 33.3 | 37.4 | <0.001 | 43.1 | 43.1 | 0.983 |
| Age (years) ^b^ | 63.2 (6.6) | 58.9 (9.0) | <0.001 | 62.2 (7.2) | 57.1 (9.5) | <0.001 | 62.7 (7.2) | 58.0 (9.4) | <0.001 |
| Height (cm) ^b^ | 158.1 (8.2) | 159.5 (8.7) | <0.001 | 158.2 (8.2) | 159.7 (8.7) | <0.001 | 159.2 (8.3) | 160.1 (8.6) | <0.001 |
| Weight (kg) ^b^ | 57.0 (10.2) | 58.7 (11.4) | <0.001 | 55.6 (9.8) | 57.5 (11.2) | <0.001 | 57.0 (10.4) | 58.0 (11.2) | <0.001 |
| Waist (cm) ^b^ | 82.0 (8.9) | 82.4 (9.8) | 0.009 | 80.5 (8.7) | 80.8 (9.5) | 0.112 | 81.1 (9.0) | 81.1 (9.8) | 0.955 |
| BMI (kg/m^2^) ^b^ | 22.7 (3.1) | 23.0 (3.4) | <0.001 | 22.1 (2.9) | 22.4 (3.2) | <0.001 | 22.4 (3.1) | 22.5 (3.4) | 0.052 |
| ABSI ^b^ | 0.0816  (0.00530) | 0.0809  (0.00520) | <0.001 | 0.0814 (0.00539) | 0.0806 (0.00524) | <0.001 | 0.0811 (0.00520) | 0.0806 (0.00514) | <0.001 |
| Diabetes category ^a^ |  |  |  |  |  |  |  |  |  |
| Normal | 89.2 | 89.4 | 0.772 | 86.2 | 84.7 | <0.001 | 84.6 | 82.5 | <0.001 |
| Borderline | 10.8 | 10.6 |  | 8.1 | 7.4 |  | 8.7 | 8.2 |  |
| Diabetes | - | - |  | 5.7 | 7.9 |  | 6.7 | 9.4 |  |
| SBP ^b^ | 128.7 (16.7) | 128.8 (18.6) | 0.663 | 120.0 (11.6) | 119.0 (12.0) | <0.001 | 128.1 (17.1) | 127.8 (18.7) | 0.378 |
| DBP ^b^ | 76.7 (10.6) | 77.3 (11.7) | <0.001 | 72.3 (8.6) | 72.1 (8.8) | 0.133 | 76.4 (10.8) | 76.5 (11.7) | 0.865 |
| LDL-C ^b^ | 128 (30.4) | 126 (32.5) | <0.001 | 129 (30.6) | 126 (32.5) | <0.001 | 112 (19.5) | 109 (20.8) | <0.001 |
| Log HDL-C ^b^ | 4.14 (0.26) | 4.12 (0.27) | <0.001 | 4.16 (0.26) | 4.12 (0.27) | <0.001 | 4.18 (0.25) | 4.16 (0.25) | <0.001 |
| Log AST ^b^ | 3.12 (0.29) | 3.11 (0.34) | 0.001 | 3.11 (0.28) | 3.08 (0.33) | <0.001 | 3.12 (0.31) | 3.11 (0.37) | 0.029 |
| Log ALT ^b^ | 2.96 (0.44) | 2.97 (0.50) | 0.200 | 2.93 (0.43) | 2.94 (0.50) | 0.381 | 2.94 (0.45) | 2.94 (0.52) | 0.800 |
| Log GGT ^b^ | 3.25 (0.65) | 3.32 (0.73) | <0.001 | 3.18 (0.62) | 3.23 (0.69) | <0.001 | 3.27 (0.69) | 3.33 (0.78) | <0.001 |
| Smoking habit ^a^ | 13.5 | 23.0 | <0.001 | 13.8 | 24.0 | <0.001 | 15.6 | 24.9 | <0.001 |

ABSI, a body shape index; WC, waist circumference; BMI, body mass index; SD, standardized difference; SBP, systolic blood pressure; DBP, diastolic blood pressure; LDL-C, low-density lipoprotein cholesterol; HDL-C, high-density lipoprotein cholesterol; AST, aspartate aminotransferase; ALT, alanine aminotransferase; GGT, gamma-glutamyltransferase

^a^ Percentage and p-value by chi-square test

^b^ Mean (standard deviation) and p-value by unpaired t-test
